# Development of salivary cortisol circadian rhythm in preterm infants

**DOI:** 10.1371/journal.pone.0182685

**Published:** 2017-08-10

**Authors:** Katrin Ivars, Nina Nelson, Annette Theodorsson, Elvar Theodorsson, Jakob O. Ström, Evalotte Mörelius

**Affiliations:** 1 Department of Pediatrics and Department of Clinical and Experimental Medicine, Linköping University, Linköping, Sweden; 2 Department of Quality and Patient Safety, Karolinska University Hospital, Stockholm, Sweden; 3 Department of Neurosurgery and Department of Clinical and Experimental Medicine, Linköping University, Linköping, Sweden; 4 Department of Clinical Chemistry and Department of Clinical and Experimental Medicine, Linköping University, Linköping, Sweden; 5 Department of Neurology, Faculty of Medicine and Health, University of Örebro, Örebro, Sweden; 6 Division of Nursing Science, Department of Social and Welfare Studies, Linköping University, Norrköping, Sweden; Istituto Superiore Di Sanita, ITALY

## Abstract

**Objectives:**

To investigate at what age preterm infants develop a salivary cortisol circadian rhythm and identify whether it is dependent on gestational age and/or postnatal age. To evaluate whether salivary cortisol circadian rhythm development is related to behavioral regularity. To elucidate salivary cortisol levels in preterm infants during the first year of life.

**Methods:**

This prospective, longitudinal study included 51 preterm infants. 130 healthy full-term infants served as controls. Monthly salivary cortisol levels were obtained in the morning (07:30–09:30), at noon (10:00–12:00), and in the evening (19:30–21:30), beginning at gestational age week 28–32 and continuing until twelve months corrected age. Behavioral regularity was studied using the Baby Behavior Questionnaire.

**Results:**

A salivary cortisol circadian rhythm was established by one month corrected age and persisted throughout the first year. The preterm infants showed a cortisol pattern increasingly more alike the full-term infants as the first year progressed. The preterm infants increase in behavioral regularity with age but no correlation was found between the development of salivary cortisol circadian rhythm and the development of behavior regularity. The time to establish salivary cortisol circadian rhythm differed between preterm and full-term infants according to postnatal age (p = 0.001) and was dependent on gestational age. Monthly salivary cortisol levels for preterm infants from birth until twelve months are presented. Additional findings were that topical corticosteroid medication was associated with higher concentrations of salivary cortisol (p = 0.02) and establishment of salivary cortisol circadian rhythm occurred later in infants treated with topical corticosteroid medication (p = 0.02).

**Conclusions:**

Salivary cortisol circadian rhythm is established by one month corrected age in preterm infants. Establishment of salivary cortisol circadian rhythm is related to gestational age rather than to postnatal age. Salivary cortisol circadian rhythm development is not related to behavioral regularity.

## Introduction

Cortisol is considered a major biomarker of stress among children and adults [1, 2]. The fetal hypothalamic-pituitary-adrenal (HPA) system responsible for cortisol release is functional by the beginning of the second trimester [3]. Cortisol is secreted in a pulsatile fashion in a circadian rhythm, peaking in the morning and displaying a nadir in the evening [4], from age one month in healthy full-term infants [5]. To further understand the development of cortisol expression in infants born preterm it is important to investigate when cortisol circadian rhythm (CCR) is established. Seven earlier studies were found investigating the development of CCR in preterm infants [6–12], three of which investigated salivary cortisol [7–9], three investigated plasma cortisol [6, 11, 12] and one serum cortisol [10]. However, there is no consensus regarding when CCR is established or whether it is dependent on gestational age (GA) or postnatal age (PNA). Kidd et al. included infants (n = 11) born before 30 weeks' gestation and found salivary CCR in single infants but not on a group level and no sustainable CCR was developed during the inclusion period, which were during the hospital stay [7]. Antonini et al. included infants (n = 9) born between 31 and 34 weeks GA and followed them longitudinal from two to 24 weeks of postnatal age. They found that salivary CCR emerged between eight and 12 postnatal weeks [8]. Vermes et al. detected plasma CCR in neonates (n = 5) from the age of 3 months [12]. The remaining four studies did not confirm CCR during the study periods, which varied between one and four postnatal weeks [6, 9–11].

Three studies have investigated factors that may influence development of CCR in infants [5, 11, 13]. Scott et al. found significantly lower cortisol levels in preterm infants requiring respiratory support and/or surfactant treatment. They also investigated CCR dependency on age and found a significant inverse relationship with higher cortisol levels at lower GA [11]. Price et al. found a weak correlation between CCR development and sleep regularity in full-term infants. Ivars et al. found a stronger CCR and behavior regularity with increasing age in full-term infants but however, no correlation between CCR development and behavior regularity as measured by the Baby Behavior Questionnaire (BBQ) [5, 14]. Trauma and depression have previously been shown to influence the HPA axis in adolescents and adults [15, 16]. But no correlation was found in full-term infants between CCR development and trauma as registered through the Life Incidence of Traumatic Events (LITE) instrument [5]. However, compared to healthy full-term infants, preterm infants are exposed to several stressful events related to intensive care in the neonatal period which, in combination with their immaturity make them more vulnerable and less resistant to additional stressful events such as family trauma. Exposure to stressful events during the neonatal period may affect the development of the HPA axis subsequently leading to altered cortisol levels during childhood [17]. Basal salivary cortisol expression across the first year of life has been found to differ by GA at birth in infants born preterm [18, 19]. Furthermore, Brummelte et al [17] found a similar pattern of cortisol across the day in preterm and full-term children at age 7 years, but a significant difference in level, with preterm infants having higher salivary cortisol levels than full-term infants at bedtime. In contrast, Buske-Kirschbaum et al. [20] found that children born preterm had higher awakening cortisol levels than those born full-term at school-age.

It is important to study when CCR emerges in infancy in preterm infants, and whether the pattern across the day differs during infancy in preterm infants compared to full-term infants. The aims of the present study were to investigate at what age CCR is established in preterm infants and to identify whether CCR is dependent on GA or PNA. Additional aims were to establish whether development of CCR is related to behavioral regularity and to elucidate salivary cortisol levels during the first year of life in preterm infants. In the present study, a control group of full-term infants was used to compare salivary cortisol levels, CCR development and development of behavior regularity [5, 14].

## Materials and methods

### Participants

A convenience sample of 51 preterm infants born at University Hospital in Linköping or at Ryhov Hospital in Jönköping were included in the study. Characteristics of the infants and their parents are compiled in Tables 1 and 2. All parents were healthy, lived together as couples, and the mothers did not use tobacco. The infants were born between GA (week+day) 23+2 and 31+6. They were divided into age groups based on GA at birth: <28 weeks and 28–32 weeks. The control group consisted of 130 healthy full-term infants (37–42 weeks GA); detailed information was presented in a recent publication [5]. All preterm infants received care in the neonatal intensive care unit (NICU) during their first weeks of life.

**Table 1:** Parental background.

Participants	Preterm infants n = 51	Control group* Full-term infants n = 130	P-value
Mothers age (years), mean (SD)	30 (5)	31 (4)	0.23
Fathers age (years), mean (SD)	31 (7)	33 (5)	0.037
**Mother’s education**			0.26
Primary education (n)	1	0	
Secondary vocational education (n)	19	22	
Secondary academic education (n)	3	6	
College/university education (n)	28	102	
**Mother’s occupation before giving birth**			0.44
Employed (n)	37	98	
Parental leave (n)	2	16	
Unemployed (n)	4	2	
Not specified (n)	8	14	
**Father’s education**			0.91
Primary education (n)	8	1	
Secondary vocational education (n)	11	28	
Secondary academic education (n)	6	5	
College/university education (n)	26	96	
**Father’s occupation**			0.78
Employed (n)	37	114	
Parental leave (n)	0	0	
Unemployed (n)	5	1	
Not specified (n)	9	16	
**Nationality of parents**			
2 Swedish parents (n)	42	122	0.38
1 Swedish parent and 1 non-Swedish European parent (n)	4	4	
1 Swedish parent and 1 non-Swedish non-European parent (n)	0	3	
1 non-Swedish European parent and 1 non-Swedish non-European parent (n)	1	0	
2 non-Swedish European parents (n)	1	0	
2 non-Swedish non-European parents (n)	0	1	
Not specified (n)	3	0	

Number of subjects (n), Standard Deviation (SD).

*Ivars et al. PLOS ONE. 2015;10(6):e0129502.

**Table 2:** Infant background: Birth, health and care.

Participants	Preterm infants n = 51
Girls/Boys (n)	31/20
Born in GA (week+day), mean (range)	28+3 (23+2–31+6)
Born in GA week; <28/>28 (n)	24/27
Firstborn (%)	51
**Antenatal steroids (Yes/No)**	**50/1**
Number of doses: one/two/three/not specified (n)	14/32/2/2
Infant born <24 h after prenatal steroids (n)	13
Infant born 24–48 h after prenatal steroids (n)	8
Infant born >48 h after prenatal steroids (n)	26
Not specified (n)	3
Birth: Vaginal/Vacuum extraction/Cesarean section (n)	21/1/29
**Infant size at birth**	
Birth weight (g), mean (SD)	1184 (406)
Birth length (cm), mean (SD)	37 (4)
SGA (%)	20
Apgar 1; 5; 10; median (Q1-Q3)	6 (4–8); 7 (6.25–9); 9 (8–10)
Age at first sampling (day) mean (SD)	22 (14)
Weight at discharge (g), mean (SD)	2671 (655)

NICU (neonatal intensive care unit). Number of subjects (n), Standard Deviation (SD), Gestational Age (GA); Small for Gestational Age (SGA); Interquartile 1–Interquartile 3 (Q1-Q3).

During their first year of life from one month corrected age (CA), 15 infants occasionally used topical corticosteroid medication (TCM) (ointment or inhalation); on average, five infants were treated each month. Other medications used to treat the infants included antibiotics, acetaminophen, non-steroid anti-inflammatory drugs, and nose drops for runny nose, as well as medication for colic and constipation. A total of 34 infants were occasionally treated with one of the non-corticosteroid containing medications described above; on average five infants each month.

None of the mothers suffered any serious diseases requiring hospitalization or intravenous drugs during the study period. All mothers except one received steroids before delivery, but thereafter only one mother was treated with corticosteroid containing medication (inhalations) during the infants first year.

### Procedure

The local ethics committee at Linköping University approved the study (D# M196-06). All babies were recruited following delivery from the maternity ward or from the NICU after oral and written informed consent was obtained from the parents.

The protocol was set up for 50 infants with 4800 saliva samples, collected three times a day, two days in a row, starting at GA week 28 until CA twelve months. A total of 4164 saliva samples were eventually collected. Sampling days were synchronized to the exact day when each infant turned GA week 28, 32, 36, 40, and thereafter at CA one month until CA twelve months; the definition of CA is well-established in the neonatal literature [21]. Based on GA at birth, the infants were included in GA week 28 or 32, mean PNA day (SD) 21.2 (13.8). To avoid any possible interference from the influence of the delivery process, the first sampling was conducted more than two days after delivery [22]. Samples were obtained on two subsequent days each month. Parents were instructed on how to take saliva samples, initially in the NICU and later at home. Samples were to be obtained no sooner than one hour after the infant slept, cried, traveled by car, or was fed solid food, and 30 minutes after intake of liquid food. Earlier studies confirm that the greatest difference in cortisol levels occurs between early morning and late evening [4]; therefore, sampling was conducted at 07:30 and 19:30, and an additional sample was taken between these times at 10:00. Sampling times ranging from 07:30–09:30, 10:00–12:00, and 19:30–21:30 were considered acceptable; samples collected outside these limits were excluded. Saliva samples collected outside the hospital were stored in the refrigerator until parents mailed them (within one week) to the University Hospital in Linköping (salivary cortisol samples are stable at room temperature for at least two weeks [23]). Once samples arrived at the laboratory they were centrifuged and stored at -70°C. A competitive radioimmunoassay was used to analyze salivary cortisol concentrations [24]. Samples were run in duplicate. Due to the numerous samples, all samples from each individual could not be analyzed in the same assay. Inter-assay coefficients of variation were 12% at 2.0 nmol/L and 6% at 10.0 nmol/L.

### Questionnaires

The BBQ, validated 1985 in a Swedish sample by Hagekull et al., measures six parameters—Intensity/Activity, Regularity, Approach/Withdrawal, Sensory Sensitivity, Attentiveness, and Manageability—based on a total of 31 items [25]. The six parameters may be considered separately or as a total score [25]. The current study measured regularity at CA one, six and twelve months, using six items: “Going to sleep at the same time,” “Waking up at the same time,” “Hungry at the same time,” “Eating the same amount of food every day,” “Taking a nap at the same time every day,” and “Having a regular bowel movement schedule.” Each item was rated on a scale from one to five, where higher scores represent higher regularity.

The LITE questionnaire was used to check for trauma [26]. The LITE questionnaire is a validated instrument that consists of 15 items with fixed answers about lifetime occurrence of traumatic life events, such as “a family member was hospitalized,” “parents separated,” and “infant hurt or threatened.” Parents were asked to fill out a Swedish version, which was used in earlier studies [5, 27], at CA one, six and twelve months.

Various possible confounding factors were monitored, including CRIB (clinical risk index for babies) score [28]. CRIB is a validated neonatal scoring system [29] that measures birth weight, GA, minimum and maximum fraction of inspired oxygen, and maximum base excess during the first 12 h, as well as presence of congenital malformations. Other possible confounding factors, monitored individually each month, included: corticosteroid medication in NICU, severe disease (defined as: infants with respiratory distress syndrome, bronchopulmonary dysplasia, patent ductus arteriosus, and/or need for surfactant treatment), days at home vs days in NICU, appropriate for gestational age (AGA) or small for gestational age (SGA) at birth, and TCM (inhalation or topical cream).

### Statistics

The software IBM SPSS Statistics, version 23 was used for statistical analysis. Statistical significance was considered if p<0.05. An independent sample t-test and Pearson chi-square were used to compare background variables between parents in this study and the control study. To investigate distribution levels in the material, the Kolmogorov-Smirnov Test was performed morning, noon, and evening; evening/morning index values. Evening/morning indexes were normally distributed, but cortisol concentrations were not.

For non-parametric statistics, the Wilcoxon rank-sum test was used to calculate potential differences between evening and morning salivary cortisol levels (for each of the 16 months). Development of CCR was defined to begin when in a specific month, infants displayed significantly higher median morning cortisol levels than median evening cortisol levels. Samples were obtained on two consecutive days each month, and prior to analysis, averages were calculated for the two morning concentrations and for the two evening concentrations for each infant and month. To avoid weighting certain individuals in the analysis higher due to large inter-individual differences in absolute cortisol concentrations, an evening/morning cortisol index was calculated for everyone by dividing the evening cortisol value by the morning cortisol value for that particular day.

A general linear model with repeated measures tested potential increase in BBQ regularity between month one, six and twelve. The hypothesis that a correlation would be present between the BBQ Regularity item and development of CCR was tested using Spearman’s correlation analysis to compare the BBQ Regularity item with the salivary cortisol evening/morning index.

An adult pattern of CCR was defined as a 20% drop in the ratio between evening and morning cortisol levels, using cut-off limits from earlier studies [5, 7, 8, 30, 31] and based on methodology accuracy.

A linear regression for repeated measurements was calculated for potential differences in development of CCR between the three different GA groups. Furthermore, a general linear model (ANOVA) was used to investigate CCR development (logarithms of evening/morning index, dependent variable) in relation to infant GA (measured in days, independent variable). The following possible confounding factors were included in the ANOVA analysis: systemic corticosteroid medication in NICU, severe disease, days at home vs days in NICU, CRIB score, AGA or SGA at birth, and temporary use of TCM in individual infants by month. The possible effect of trauma (measured by LITE) on development of CCR was tested using Spearman’s correlation analysis to compare the number of traumas with the salivary cortisol evening/morning index.

### Outliers

The material included 23 extremely high cortisol values >1000 nmol/L. Two infants were accounted for 18 of these values between month five to nine and an additional four infants had occasionally (month 2, 7, 8, and 10) a salivary cortisol value >1000 nmol/L. These outlier values are all included in the statistical analyses but excluded when mean values for salivary cortisol levels during the first year of life is presented.

### Missing data

**Fig 1:**
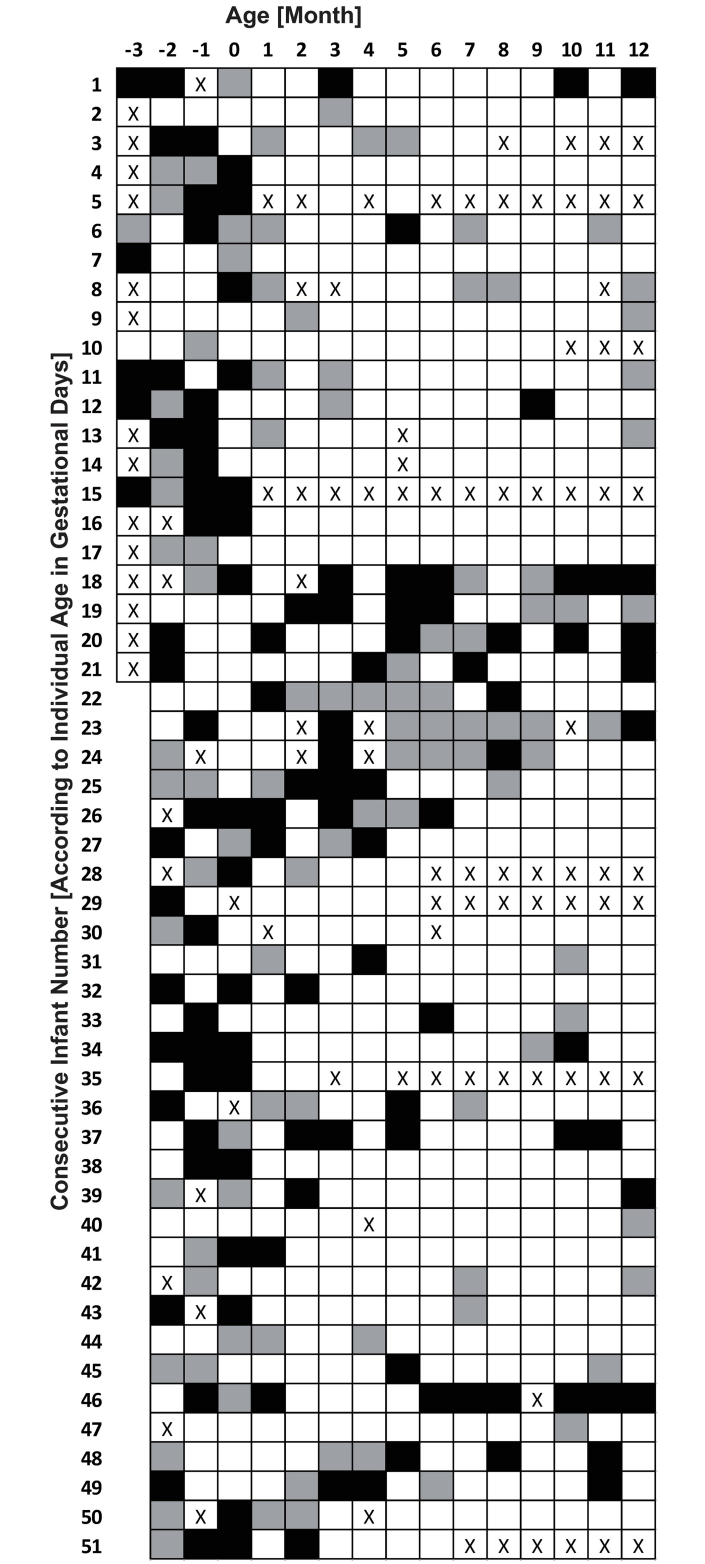
Individual CCR development. Each square: Index for one infant, one month, White—“CCR positive” (evening/morning cortisol <0.80 equal to an established CCR), Black—“CCR negative” (evening/morning cortisol >1.20 equal to a non-established CCR), Gray—neither “positive” nor “negative” (evening/morning cortisol 0.80–1.20). X-squares: “Missing Index”. Month: -3, -2, -1 and 0 = gestational week 28, 32, 36 and 40.

Reasons for missing cortisol samples include: severe illness preventing sampling, one infant died due to severe illness, failure of parents to send samples to hospital, insufficient quantity of saliva, and samples obtained outside allotted time slots. In total, 636 (13.3%) of 4800 samples were missing. The missing evening/morning indexes are marked with an X for each month in Fig 1.

## Results

**Fig 2:**
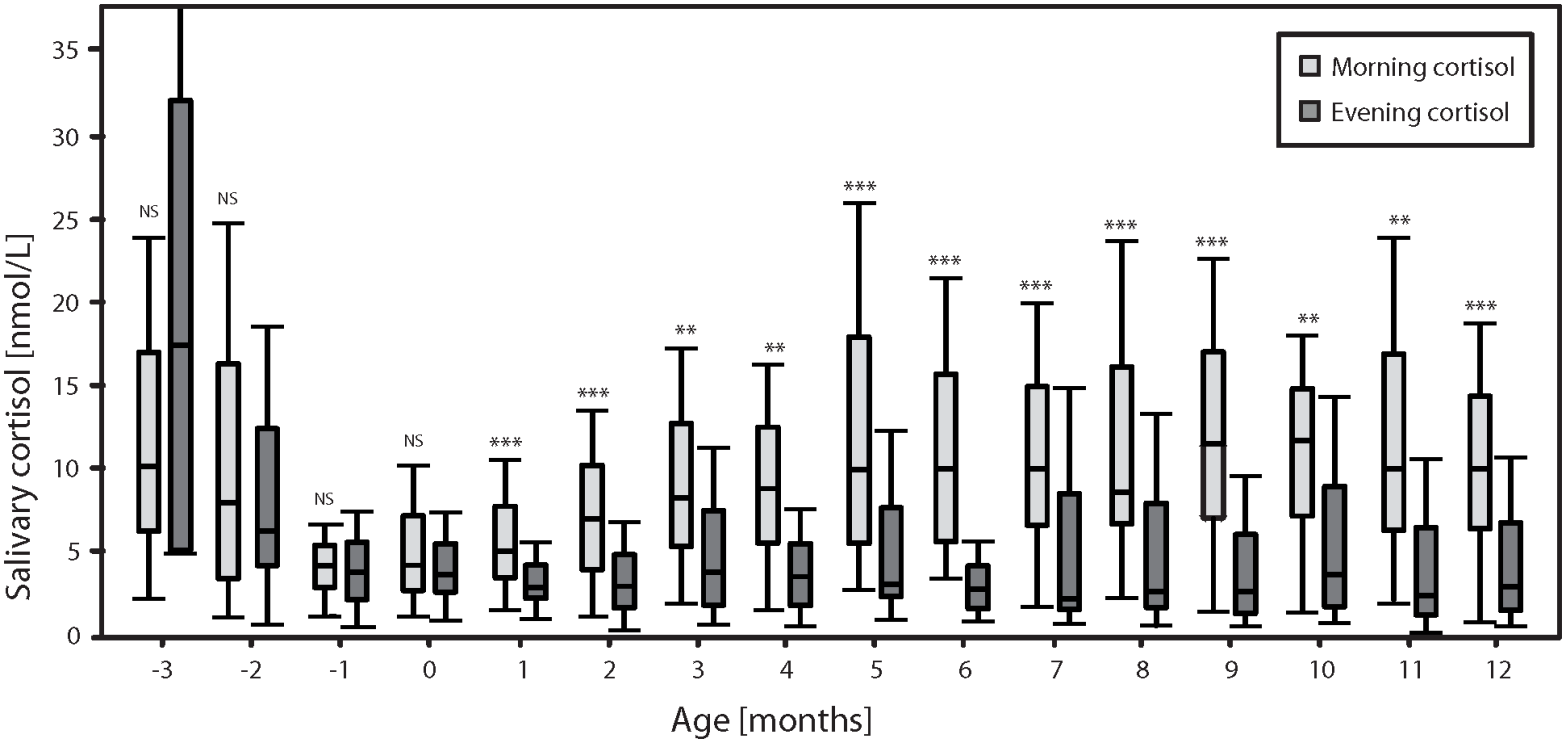
Boxplots. Light grey = morning, dark grey = evening; monthly median, inter-quartile range one and three. *** = P < 0.000, ** = P < 0.00. Wilcoxon’s rank-sum test, month minus three to twelve, (month: -3, -2, -1 and 0 = gestational week 28, 32, 36 and 40).

On a group level from one month CA, preterm infants developed CCR—defined by significantly higher morning cortisol levels than evening cortisol levels—that persisted throughout the first year of life CA, Fig 2; Table 3.

**Table 3:** Monthly difference between evening and morning salivary cortisol levels.

Corrected age at sampling, (Month)	Postnatal age at sampling, days Mean (SD)	Subjects with analyzable samples (n)	Difference evening/morning cortisol level P-value
-3	15 (9)	7	0.043*
-2	26 (16)	45	0.569
-1	53 (15)	46	0.723
0	81 (16)	49	0.574
1	111 (17)	48	0.000
2	140 (16)	45	0.000
3	172 (18)	48	0.003
4	202 (17)	45	0.001
5	232 (18)	47	0.000
6	261 (18)	45	0.000
7	293 (18)	45	0.000
8	321 (18	44	0.000
9	351 (19)	44	0.000
10	381 (20)	42	0.001
11	411 (19)	42	0.001
12	441 (20)	43	0.000

Monthly p-values for difference between evening and morning cortisol on a group level using Wilcoxon’s rank-sum test. Age for sampling measured in: Corrected Age Months, minus three to twelve, (month: -3 = gestational week 28, -2 = gestational week 32, -1 = gestational week 36 and 0 = gestational week 40, 1 = gestational age week 40 plus one month and so on monthly until month twelve). Postnatal Age for sampling measured in: Mean Day for sampling during preterm infants’ first year of life. Number of infants that submitted analyzable samples for evening/morning index in each month. Statistical significance was considered if p<0.05.

*The p—value in month -3 shows a statistically significant difference between evening and morning cortisol levels, but the higher and lower levels are reversed with respect to CCR (evening cortisol level > morning cortisol level).

Fig 1 presents individual patterns of CCR development in preterm infants during the first year of life; for reference values from control group see [5]. The following patterns were observed: CCR developed at an early age and persisted each month thereafter; some infants failed to develop a stable pattern, deviating occasional months from CCR at an older age; and some infants failed to develop any CCR during the first year of life, as defined by the cut-off of a 20%-difference.

Evening/Morning mean indexes by group were: Preterm infants GA <28 weeks = 0.722, Preterm infants GA 28–32 weeks = 0.642, and Controls GA 37–42 weeks = 0.563. There was a significant difference between each of the two groups of preterm infants and the control group (p = 0.00 and p = 0.00, respectively), but not between the two preterm infant groups.

Development of CCR in relation to GA, measured in days, showed that CCR increased with higher GA. Infants with higher GA developed CCR at a lower PNA than infants with lower GA. CCR development was dependent on infant GA rather than PNA. The decrease in evening/morning cortisol index was 0.02 per day of increased GA, counted in days, for the same PNA.

The BBQ Regularity item score increased significant between CA month one and six (p = 0.000), and six and twelve (p = 0.000) (Table 4). No significant correlation was observed between the BBQ Regularity item score and development of CCR, in line with the results from the full-term control group [5, 14].

**Table 4:** “Regularity” measured with the Baby Behavior Questionnaire.

BBQ Regularity			
**Month**	**1**	**6**	**12**
**Mean**	2.7	3.6	3.9
**SD**	0.9	0.7	0.6
**Difference in p-value**		0.000*	0.000**

*P-value for difference between month one and six corrected age.

**P-value for difference between month six and twelve corrected age. Scale 1–5 where increased numbers represent increased regularity.

Standard deviation (SD), Baby Behavior Questionnaire (BBQ).

Table 5 presents median morning, noon, and evening cortisol levels. The highest variability in cortisol levels occurred at GA weeks 28 and 32. Stabilization began at GA weeks 36 and 40. To show inter-individual variability in absolute basal levels for preterm infants Table 5 also presents mean morning, noon, and evening cortisol levels.

**Table 5:** Baseline salivary cortisol levels for preterm infants.

Age Month	Morning values: 07:30–09:30					Noon values: 10:00–12:00					Evening values: 19:30–21:30				
	Median	Q1-Q3	Min-Max	Mean	SD	Median	Q1-Q3	Min-Max	Mean	SD	Median	Q1-Q3	Min-Max	Mean	SD
**-3**	10.0	6.2–16.7	2.3–19.7	11.1	6.7	11.3	7.1–14.8	2.0–32.7	12.5	9.6	17.1	5.0–31.5	4.9–56.8	21.5	19.5
**-2**	7.8	3.4–16.0	1.2–152	17.8	27.8	6.0	3.5–13.1	1.3–197	14.1	30.3	6.2	4.2–12.2	0.8–77.8	12.1	16.8
**-1**	4.2	2.9–5.4	1.2–23.7	4.8	3.6	3.8	2.8–5.5	1.0–32.1	5.2	5.4	3.8	2.2–5.5	0.6–28.8	4.6	4.3
**0**	4.2	2.7–7.1	1.2–17.5	5.4	3.8	4.1	2.6–6.1	1.6–24.3	5.5	4.5	3.6	2.6–5.4	1.0–29.5	5.1	5.1
**1**	5.0	3.4–7.6	1.6–78.7	7.6	11.0	4.0	3.0–6.5	1.6–34.9	5.8	5.3	2.9	2.2–4.2	1.1–100	5.7	14.3
**2**	6.9	3.9–10.1	1.3–153^#^	10.6^#^	22.1^#^	4.6	3.2–7.6	1.0–191	10.2	27.7	2.9	1.7–4.8	0.4–352	14.5	53.5
**3**	8.2	5.3–12.5	1.9–125	13.1	19.3	5.9	3.3–12.0	1.2–178	14.0	28.7	3.8	1.8–7.4	0.7–145	10.0	23.4
**4**	8.7	5.5–12.3	1.6–126	14.8	24.5	6.9	5.0–8.7	1.4–516	29.0	102	3.5	1.8–5.5	0.6–249	16.7	49.9
**5**	9.8	5.5–17.6	2.8–651^#^	37.0^#^	105^#^	6.1	4.5–10.5	1.8–747^#^	36.9^#^	128^#^	3.1	2.3–7.6	1.0–225^##^	15.5^##^	44.4^##^
**6**	9.9	5.6–15.4	3.4–23.9^##^	10.8^##^	5.7^##^	5.1	4.2–6.8	1.6–16.9^##^	5.7^##^	2.8^##^	2.8	1.6–4.1	0.9–405^#^	12.1^#^	58.6^#^
**7**	9.8	6.5–14.7	1.9–390^##^	29.9^##^	72.4^##^	5.3	3.2–9.2	2.0–366^##^	18.3^##^	56.3^##^	2.2	1.6–8.4	0.8–476^##^	22.2^##^	75.5^##^
**8**	8.5	6.6–15.8	2.3–487	40.2	93.2	5.9	4.5–10.1	1.5–815^#^	32.8^#^	122^#^	2.7	1.7–7.8	0.6–204^#^	17.4^#^	43.1^#^
**9**	11.3	7.0–16.7	1.5–362^##^	39.3^##^	90^##^	4.8	3.4–7.8	0.9–494^##^	34.6^##^	103^##^	2.6	1.3–6.0	0.6–328	27.9	72.8
**10**	11.5	7.1–14.6	1.6–206	23.6	38.9	5.0	2.9–9.0	1.1–150^#^	14.9^#^	31.2^#^	3.6	1.7–8.8	0.9–813	37.7	134
**11**	9.9	6.3–16.6	2.1–530	28.6	81.0	4.9	2.9–10.7	1.9–209	17.6	39.0	2.4	1.3–4.4	0.2–311	22.6	60.3
**12**	9.9	6.3–14.1	1.0–68.2	14.3	15.0	4.5	3.1–9.0	0.6–103	12.2	20.8	2.9	1.5–6.7	0.6–141	11.5	28.5

Monthly median (quartile 1 –quartile 3), min-max values, and mean (standard deviation) salivary cortisol levels [nmol/L] at three different sampling times: morning (07:30–09:30), noon (10:00–12:00) and evening (19:30–21:30). Corrected Age in months: minus three to twelve, (month: -3 = gestational week 28, -2 = gestational week 32, -1 = gestational week 36 and 0 = gestational week 40).

^#^ = without one outlier >1000 nmol/L

^##^ = without two outliers >1000 nmol/L

Development of CCR in preterm infants was not affected by the following possible confounding factors: number of days spent in hospital or at home after birth, CRIB score, AGA or SGA at birth, severe disease, or corticosteroid treatment during NICU care. TCM as a confounding factor was associated with higher morning, noon, and evening cortisol levels in individual infants for each single month of medication use (p = 0.02). Development of CCR in infants treated with topical corticosteroid during occasional intermittent months was delayed by a statistically significant difference (p = 0.02).

No significant correlation was found between single trauma (LITE) and cortisol levels. The number of traumas was insufficient to allow testing for possible multiple trauma influences (Table 6).

**Table 6:** Number of reported traumas at three different time periods during infants’ first year of life.

Number of Traumas (n)	CA Month 1 Number of infants (n)	CA Month 6 Number of infants (n)	CA Month 12 Number of infants (n)
**1**	6	6	5
**2**	7	4	0
**3**	1	0	0
**Total**:	14	10	5

Corrected age (CA). The traumas were most often hospital-related, which resulted in reported traumas in a larger number of infants at a lower age due to hospitalization for prematurity and birth-related sickness. Trauma was otherwise rare in the group. The most common trauma other than personal hospitalization included hospitalization of a family member (most often mother).

## Discussion

The current results, based on 4164 samples of salivary cortisol from 51 preterm infants, show that CCR is established on a group level by the age of one month CA. It persists throughout the first year and is dependent on GA rather than PNA. Moreover, the study presents consecutive salivary cortisol levels for preterm infants from birth until age twelve months CA.

Seven earlier studies have investigated development of CCR in preterm infants [6–12]. Regardless of whether the source of cortisol was saliva, plasma, or serum, the results from these studies were either in line with ours regarding time for onset of CCR [8, 12], or were not designed to cover that time period (one month CA) [6, 7, 9–11]. However, most previous studies only report on the first observed CCR age but not what happens later, after the first detected CCR. One strength of the present study is the longitudinal design showing that CCR can develop earlier or later in individuals but that once observed does not mean it is stable.

It is known that maturation of the adrenal cortex in preterm infants relates to GA (in infants born before GA week 33) [32]. Low GA in preterm infants is often used to predict perinatal morbidity, delayed maturation of lung function, and state of health, as well as illness and concentration difficulties later in life [33, 34]. Only Scott et al. have previously considered development of CCR in relation to GA or PNA [11]. Their results show an inverse relationship, with higher cortisol levels at lower GA and CCR dependency on GA. Our current results show that CCR develops in preterm infants at one month CA and confirm that CCR is dependent on GA. CCR at one month CA in preterm infants resembles CCR at one month PNA in full-term infants [5]. The present study adds important knowledge on this subject; to our knowledge, it is the most extensive in the literature with regard to sampling regularity, consistency, and duration.

An increased regularity (BBQ) developed during the first year of life in preterm infants but no significant correlation was seen between CCR development and regularity either in our preterm material or in full-term infants [5, 14]. Sleep-wake patterns and regularity have previously been studied in relation to CCR development in preterm [8] and full-term infants [5, 13, 14]. Sleep time increased at PNA week eight in both studies, but there was no correlation with CCR. The observed lack of correlation between CCR and behavioral regularity in this and prior study [5, 14] may be because CCR depends on maturity of biological functions such as maturation of the adrenal cortex and other developmental functions that accompany increased GA [32], whereas regularity in behaviors such as sleep seems to be dependent on both GA and environmental influences, e.g. days at home or in hospital care [35].

Due to large inter-individual variations in absolute morning-evening cortisol differences, the Wilcoxon rank-sum test was selected to evaluate the main parameters in the current study as well as in earlier studies [7–9, 30, 31]. Also, several previous studies have corroborated that absolute basal cortisol levels vary substantially both intra-individually and inter-individually [7, 10–12, 19, 36–38], which is why an evening/morning cortisol index for each individual was considered more appropriate when analyzing data from multiple individuals in the current study. It is also well-known that preterm infants have greater variability and higher cortisol levels than healthy full-term infants [7, 10–12, 19, 36, 37]. Except from large individual variations in absolute cortisol levels there were also some extreme outliers in the present study. Two infants were accounted for 78% of the values >1000 nmol/L. One explanation for this could be treatment with TCM. TCM has previously been shown to affect cortisol levels in preterm infants with respiratory diseases [39, 40]. The present study confirms these results, showing higher cortisol levels both morning and evening, and in addition presents new results showing an association between TCM and delayed CCR development. No prior study on CCR development in preterm infants has reported this important finding [6–12], which signals the need for further studies on this matter.

No correlation was found between trauma (LITE) and cortisol levels, which is in line with the results on healthy full-term infants [5], but in contrast to another study where multiple traumas in abused adolescents did influence salivary cortisol levels [15]. One plausible explanation for this lack of significant correlation may be that most of the infants experienced few traumatic life events other than severe illness and hospitalization.

Hospitalization in a NICU often involves separation from the mother that may affect the development of the HPA axis. For instance, parental closeness and skin-to-skin contact increase the co-regulation of salivary cortisol levels between preterm infants in the NICU and their mothers while separation seems to delay such co-regulation [41, 42]. This needs to be further investigated in relation to CCR in future studies.

### Limitations

There are some limitations with the study; the sample size is more limited than the control group of full-term infants and some infants were too ill at the beginning of the study to be eligible for saliva sampling; a larger population would have contributed to more data regarding cortisol levels in early preterm infants. Another limitation is sampling frequency. Cortisol levels peak in the morning and reach a nadir in the evening. Our sampling times were morning, noon, and evening, but an entire 24-hour cortisol pattern would perhaps have yielded even more information about CCR, especially in hospitalized preterm infants in the NICU environment. Neither did we collect information about the time of awakening in relation to each saliva sampling because of the risk with accuracy of data due to infants’ different sleep patterns and sleep durations during the first year. Instead we focused on the regularity.

## Conclusions

A salivary cortisol circadian rhythm in preterm infants can be detected—on a group level—at one month corrected age, which is earlier than previous studies have shown. The development of the salivary cortisol circadian rhythm is dependent on gestational age rather than postnatal age. The current study also provides never-before-published data on consecutive salivary cortisol levels in a fairly large population of preterm infants from birth until twelve months corrected age. Topical corticosteroid medication was found to be associated with elevated levels of cortisol and significantly delayed the development of the salivary cortisol circadian rhythm.

## Abbreviations

AGA: appropriate for gestational age
BBQ: Baby Behavior Questionnaire
CA: corrected age
CCR: cortisol circadian rhythm
CRIB: Clinical Risk Index for Babies
GA: gestational age
HPA: hypothalamic-pituitary-adrenal
LITE: Life Incidence of Traumatic Events checklist
NICU: neonatal intensive care unit
PNA: postnatal age
SGA: small for gestational age
TCM: topical corticosteroid medication
